# "The lobbying strategy is to keep excise as low as possible" - tobacco industry excise taxation policy in Ukraine

**DOI:** 10.1186/1617-9625-8-10

**Published:** 2010-08-31

**Authors:** Konstantin S Krasovsky

**Affiliations:** 1 Alcohol and Drug Information Centre (ADIC-Ukraine), Vishnyakovskaya Str., 13-212, Kyiv, 02140, Ukraine

## Abstract

**Background:**

Tobacco taxes are one of the most effective ways to reduce tobacco use. Transnational tobacco companies (TTCs) claim they wish to develop and secure excise systems that benefit both governments and the profitability of the companies themselves. The objective of the paper is to use the case of Ukraine, with its inconsistent history of excise tax changes in 1992-2008, to explore tobacco industry taxation strategies and tactics, and their implications for governmental revenues.

**Methods:**

Details of tobacco industry policy on tobacco taxation in Ukraine were obtained by searching tobacco industry internal documents and various published reports.

**Results:**

Even before entering the market in Ukraine, TTCs had made efforts to change the excise system in the country. In 1993-1994, TTCs lobbied the Ukrainian Government, and succeeded in achieving a lowering in tobacco tax. This, however, did not produce revenue increase they promised the Government. In 1996-1998, Ukrainian authorities increased excise several times, ignoring the wishes of TTCs, caused significant growth in revenue. Due to TTCs lobbying activities in 1999-2007 the tax increases were very moderate and it resulted in increased tobacco consumption in Ukraine. In 2008, despite the TTCs position, excise rates were increased twice and it was very beneficial for revenues.

**Conclusions:**

The Framework Convention on Tobacco Control includes provisions both on tobacco taxation policy and on protection of public health policy from vested interests of tobacco industry. This paper provides arguments why tobacco taxation policy should also be protected from vested interests of tobacco industry. TTCs taxation strategy appears to be consistent: keep excise as low as possible. Apparent conflicts between TTCs concerning tax structures often hide their real aim to change tax structures for competing interests without increasing total tax incidence. Governments, that aim to reduce levels of tobacco use, should not allow tobacco companies to influence the development and implementation of tobacco taxation policy.

## Background

Tobacco taxation is the most effective way to reduce tobacco use, especially among young people. As an additional benefit for governments, tax increases provide increased revenues [1]. When the Soviet Union collapsed each of the newly independent states introduced some kind of market reform. For the tobacco industry, market reform meant that state-controlled tobacco enterprises could be privatised and tobacco excise taxation should be introduced. Several publications [2-5] described the process of tobacco industry privatisation in the Former Soviet Union countries by transnational tobacco companies (TTCs) with widespread implications for tobacco control across the world.

TTCs investments in Ukraine started in 1993, when the British-American Tobacco (BAT) acquired 65% of the Priluky tobacco factory. A detailed description of these investments is presented in [6]. Most major TTCs (BAT, Philip Morris, RJR-JTI and Imperial Tobacco-Reemtsma) are present in Ukraine and, since 1994, they control more than 90% of tobacco market. In Ukraine, manufactured cigarettes make up 99% of tobacco market.

Tobacco taxation policy is a key element of an evidence-based and comprehensive national tobacco control programme. The Framework Convention on Tobacco Control (FCTC) states that price and tax measures are an effective and important means of reducing tobacco consumption. The FCTC also urges that in setting and implementing their public health policies with respect to tobacco control, Parties shall act to protect these policies from commercial and other vested interests of the tobacco industry.

TTCs claim they have detailed expertise on tobacco taxation and can help governments to establish optimal excise rates. In 1993, BAT's managing director Ulrich Herter in his speech to the World Tobacco Symposium in Moscow, claimed: "*It is a fact, little appreciated outside the industry and the governments we deal with that, in addition to all our other skills, we engage in one of the world's "oldest professions", tax collection. Not only do we collect excise but we also advise and set up schemes in many countries, that will deliver to their governments the revenues they need in a predictable and orderly way*" [7]. In 1998 the BAT claimed its aim was: "*to develop and secure excise systems that benefit both governments and the company*" [8].

Back in the mid-1980 s, excise taxes became an increasingly urgent issue for the tobacco industry, leading it to formulate strategies and to assemble arguments to oppose excise tax increases [9]. Research revealed that TTCs managed to persuade governments to keep low tobacco taxes in Finland,[10] Hungary,[11] Uzbekistan [12] and other countries.

The objective of the paper is to use the case of Ukraine with its inconsistent history of excise tax changes in 1992-2008 to explore how the tobacco industry seeks to advance its vested interests through involvement in the development of tobacco taxation policies, and in the implications for government revenues.

## Methods

An understanding of the tobacco industry's interest in tobacco taxation in Ukraine was obtained by searching in 2003-2009 the tobacco industry internal documents at http://tobaccodocuments.org and http://legacy.library.ucsf.edu. The methods have been described in detail in [2-5,13], and consisted primarily of online searching using search terms including Ukraine, Ukr, tax, excise and others in a broad and iterative approach. Initially several thousand documents were found, but to make the search more specific the following categories of documents were considered as ineligible: 1) documents with information on excise in other countries, but other issues in Ukraine; 2) documents on excise in other countries, where Ukraine is used just for comparison; 3) excerpts of legislation and other documents where tax rates and regulations are just presented; 4) newspaper articles, where tobacco industry position is not mentioned. Finally the search retrieved 240 files (many of which were duplicates), which had some information on tobacco industry positions and attitudes to tobacco taxation. From 240 files after excluding all kinds of duplicates (translations into Ukrainian, several versions of one document, etc) over 60 documents were studied in detail. Documents were indexed in a database which enabled the sorting of documents by date and topic to construct a historical and thematic narrative. Supplementary data were obtained from the tobacco control media monitoring database, which was established in 2000 as part of activities of the Tobacco Control Resource Centre for the Russian-speaking countries (see http://www.adic.org.ua/adic), statistical data and other published reports.

To present the outcomes of the taxation policy variations, inflation-adjusted revenue levels based of the official statistics data were calculated.

## Results

### 1992: Creating the taxation climate prior to starting investments

In 1992, just before starting investments in Ukraine, BAT manager Paul Brigham issued an internal document titled "*Key Area Paper. Excise Taxation of Tobacco Products*",[14] where he set out BAT's taxation policy aim *"To manage the cigarette excise taxation issue in order to establish the most appropriate retail price structure whilst optimising profitability and cash flow over the long term"*. This policy was set out to be achieved by focusing upon several main strategies. The first strategy was: "*Seek to reduce excise taxation or to ensure that its level does not increase at a rate greater than inflation*". One of the arguments supporting this strategy was that "*increases in taxation may result in a decline in consumption sufficient to bring about a fall in revenue yield to the Government"*. However Brigham himself questioned this argument "*Because of the inelasticity of demand for cigarettes over the longer term, this result rarely occurs*". In 1993 Philip Morris (PM) formulated an almost identical taxation strategy to "*Seek to minimize the total tax burden on cigarettes in all instances*" and "*ensure that retail price increases do not exceed inflation*" [15].

Before starting investments in Ukraine, BAT developed a detailed business plan. It was based on market estimates in 1991 of a total Ukrainian cigarette market of about 65 billion cigarette, while the estimated market potential was 80-85 billion cigarettes [16]. BAT manager Anton van Waay commented: "*If this market demand is not satisfied over a longer period, it should be questioned whether this theoretical demand will not disappear" *and to satisfy the demand "*what is needed is a strategy for excise and tax" *[17]. He also indicated that "*cigarettes are extremely cheap in Ukraine" *[18] and suggested "*Would it not be better to have a strategy aimed at a low excise on plain, a mix structure of specific/ad valorem a la West Europe, but a **lower total tax burden**?" *[19]. The business plan for Ukraine stated "*As a precondition to BAT's involvement in the Ukraine, the excise system must be change*d" [16] Another document reports about the meeting to discuss "*whether the authorities would be amenable to an approach to discuss and advise on excise structures" *[20]. At the meeting it was concluded that "*it is essential that there are excise systems within which we can work, and it is strongly preferable that they should provide a Framework within which we can make a profit*".

While requiring changes to the excise taxation system, TTCs actually seemed ready to invest without such changes [21]. BAT managing director Herter wrote "*Excise structure changes are necessary and whilst attempts will be made to change the structure immediately, it is recognised that it might not be possible to effect such changes before an acquisition takes place*" [22].

### 1993-1994: successful lobbying of tax rates decrease

Just before the TTCs acquisition of the first tobacco factory in Ukraine, excise taxes on cigarettes were increased to 70% of ex-factory price both for filter and non-filter cigarettes. Since May 1992, when the BAT Business Plan was prepared, the prices of cigarettes have doubled in real terms [23]. BAT manager Anne Johnson commented: "*Although the increase in excise rates is substantial, the rates are now not substantially different from those in neighbouring countries*" [23]. She also mentioned: "*The new excise rates will have an impact on the anticipated volumes and therefore on the profitability of the project. However, overall the project remains financially extremely attractive*".

In June 1993, BAT's chairman Patrick Sheehy wrote a letter to the President of Ukraine, Leonid Kravchuk,[24] where he described his perspective on the tobacco market in Ukraine. "*At the present time, cigarette production has declined by some 50%. The consequence of this is that consumer demand for cigarettes is not being satisfied. The inevitable result is that the Republic's revenue from excise and tax is not meeting the forecasts"*. Mr. Sheehy indicated that "*This situation is attributable to the amount and structure of the excise introduced this year... overall total burden or level of taxation on cigarettes is high with the result that cigarettes are becoming unaffordable to the majority of consumers in Ukraine"*. Then Mr. Sheehy quite forthrightly suggested to the President of Ukraine "*I would therefore suggest that the **amount of excise be re-examined and lowered"***. And it was not just a suggestion, but a submitted proposal, as Sheehy wrote: "*I have been informed that the Ministry of Food, which is responsible for the tobacco industry, has already submitted detailed proposals for a change in the excise structure. I would, Mr. President, be most grateful if you would support these proposals for a change in excise so that the difficulties which the industry is currently facing in Ukraine can be alleviated as soon as possible"*. These proposals were developed with the assistance of the author of BAT's taxation strategy [23].

**Figure 1:**
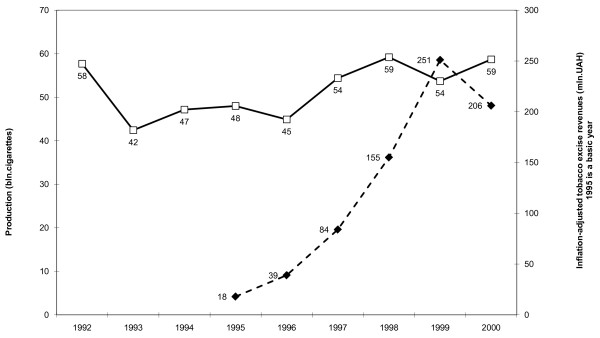
**Cigarette production and revenues in Ukraine in 1992-2000**. Production (bln. cigarettes). Inflation-adjusted tobacco excise revenues (mln.UAH) 1995 is a basic year.

The BAT proposal seemed to be favourably received. In December 1993, the government lowered tax rates on filtered cigarettes to 60%, and the non-filter cigarette rate to 45% [6]. Since 1994, only the Parliament of Ukraine has the powers to change tax rates, while the government and Members of Parliament (MP) may submit their proposals on changing the rates. In February 1994, the Parliament lowered excise rates to 50% and 35% respectively. But even this turned out to be too little, and in October 1994 the rates were adopted at a level of 40% for filtered cigarettes and 10% for non-filter cigarettes. Production of cigarettes in Ukraine increased from 42 billion sticks in 1993 to 48 billion sticks in 1995 - only a 12% increase (Fig. 1). Such a small increase could not compensate revenue losses through the lowering of tax rates [6].

### 1995-1998: Ukrainian authorities were increasing tobacco tax rates, ignoring tobacco industry positions

In 1995 the Ukrainian authorities recognized that meeting TTCs taxation proposals did not provide the promised results. Then TTCs used a front group to intervene in taxation policy in Ukraine. Back in 1992 it was decided to "*establish excise advisory team to offer advice to Governments; possibly using Deloitte assistance" *[25,26]. In September 1995, professional services company Deloitte and Touche issued a report in Ukrainian titled "Research of possible ways to increase State budget revenues from tobacco products taxation in Ukraine" [27,28]. The report's proposals were to increase rates for non-filter cigarettes from 10 to 20%, while decreasing rates for filter cigarettes from 40 to 20%. The report promised that with such rates TTC would increase their investments to filter cigarette production and eventually it would provide the equivalent of an additional $174 million (US) in excise revenues for the next 5 years.

In February 1996, the Parliament imposed taxes on filtered cigarettes at the level of 2 ECU (European Currency Unit was used before the euro) per 1000 cigarettes, and for non-filter cigarettes the tax was temporarily set at 0.5 ECU in 1996 and 1 ECU in 1997. This meant that excise duty increased approximately 1.3-fold for filtered cigarettes and 2-fold for non-filter cigarettes [6].

In July 1996, the Ukrainian Tobacco Association claimed that "*the adopted rate level destroys the domestic tobacco industry" *and proposed to decrease the level of excise duty both for filtered and non-filter cigarettes [6]. This proposal was substantiated with the expectation that an increase in filtered cigarette production would allegedly increase aggregate budget revenues.

However due to increases in excise rates, budget revenues from tobacco taxation in 1996 totalled 53 million Ukrainian Hryvnas (UAH) compared to annual projection of 23 million UAH. For the four year period of tax increases over 1996-1999, the total revenue from tobacco excise was $342 million (US) which was almost $300 million (US) more than would have been raised under the old taxation system, while Deloitte and Toche's proposals promised only $174 million (US) for five years.

In 1996-1998, TTCs mainly tried to stop additional tax rate increases. In 1996, the tax rate for plain (non-filter) cigarettes was 4 times lower than for filter cigarettes and the BAT objective on excise was to *"influence the Government and Parliament to hold the excise tax for plain cigarettes in line with the low purchasing power of the rural consumer" *[29]. The RJR document [30] reports, that in October 1997: "*Proposals of the Ukrainian government to incorporate an additional increase of excise tax and customs duty for all kinds of tobacco products by 0.5 ECU were rejected by the parliament. The position of the industry was taken into account"*.

In 1998, Government Ministries had different positions on tobacco taxation. A PM document [31] indicated: *"The Ministry of Finance proposes to retain the current fully specific one tier structure and to increase the rate from 2 to 3 ECU per thousand cigarettes. The Ministry of Foreign Economic Relations and Trade proposes to make no changes to excise rate or structure but, instead, to increase the import duty from 2.5 to between 3 and 3.5 ECU per thousand cigarettes. The Ministry of Economy proposes to retain the existing structure and existing rate levels for both excise taxes and import duty. Area continues to support the Ministry of Economy proposals"*. As usual, PM supported the lowest rates.

TTCs could have different positions on tax rates depending on their brand portfolio. TTC Reemtsma, which was the main producer of non-filter cigarettes, proposed to decrease non-filter cigarettes rates from 2 to 1.5 ECU, while to increase filter cigarettes rates from 2 to 2.5 ECU, however "*the Ministries did not support the proposal because it would lead to a substantial reduction of state budget revenue*" [31].

In June 1998 the Government proposed to increase excise rates for tobacco products. BAT meeting with PMI and RJR, representatives discussed the steps to be taken by tobacco companies. They prepared "*information based on economic calculations, which will show that such a decision will not lead to big contributions to the budget but, on the contrary, will lead to increase contraband and breakdown of Ukraine's tobacco industry*" [32].

However, in August 1998 tax rates were increased to 3 ECU for filter cigarettes and 2.3 ECU for non-filter cigarettes. But in two weeks the notorious August 1998 fiscal crisis occurred and tax rates (in UAH) almost doubled. That is why in December 1998, the parliament changed the rates to 2.5 ECU per 1000 cigarettes. In 1998, excise rates per 1000 cigarettes increased from 4.2 UAH in January to 10 UAH in December. The nominal government revenues increased to 129 million UAH in 1997 and 287 million UAH in 1998 [6]. Government again had got a benefited by rejecting tobacco industry proposals on tobacco taxation reform.

In 1999, the Parliament adopted new laws to introduce a special 5% tax on cigarettes earmarked for the country's Pension Fund. PM's documents [33,34] demonstrate a negative tobacco industry attitude towards this law. However, the tax was in place until 2004 and contributed some 700 million UAH to the Pension Fund.

During 1996-1998 the Ukrainian authorities, despite apparent tobacco industry resistance, increased tobacco excise rates several times and every time it was very beneficial for the tax revenue. In real (inflation-adjusted) terms, tax revenues in 1995-1999 increased by 14 times, while production increased only by 12% (Fig. 1). In 1997-1999 cigarette price index exceeded the inflation rate, so the real price for cigarette became higher and higher. Over this period, demand for tobacco products decreased, with clear benefits for public health in Ukraine.

### 1999-2000: Tobacco industry managed to stop tax rate increases

In 1999, tax rates for non-filter cigarettes were much higher in Ukraine than in neighbouring Russia which prompted bootlegging of Russian cigarettes to Ukraine. The TTCs in their public statements intentionally exaggerated amounts of smuggled cigarettes [6] and made reference to the situation to lobby tax rate reduction [35].

According to the BAT CORA's (Corporate and Regulatory Affairs unit) Budget Proposals for 1999, $140,000 (US) were allocated for lobbying on tobacco taxation in Ukraine [36]. A seminar for senior government officials was also organised to present BAT's proposals on tobacco tax [37]. BAT in Ukraine also organised a visit for Ukrainian ministers, parliamentarians and customs officials to the UK and Germany to examine tax structures in those countries [38].

In November 1999, the Parliament introduced the tobacco tax rate in local currency - 10 UAH per 1000 filter cigarettes and 7 UAH per 1000 non-filter cigarettes. Actually, the tax was reduced from 2.5 ECU both for filter and non-filter cigarettes to 2.07 ECU for filter cigarettes and 1.45 ECU for non-filter cigarettes. The period of tobacco tax rates increases was over although excise revenues in 1999 reached 522 million UAH. In 2000, despite cigarette production increases, only 444 million UAH was raised from tobacco excise [6]. In real terms, revenues (including the Pension Fund payments) decreased by 20% (Fig. 1).

In 2000, BAT developed excise policy proposals that suggested maintaining a stable tax burden for 3-5 years, and before starting proposed a "period of stability" during which the excise rate should be reduced to 8.0 UAH per 1000 cigarettes [39]. However the Government proposed increasing excise rates from 10 to 13.5 UAH. BAT conducted successful lobbying to keep the rate [40].

### 2000-2007: Fighting around excise structure to prevent excise increase

In 2000-2007, the total excise incidence changes were small but the structure of excise was changed several times. Those TTCs that mainly produce cheap cigarettes (BAT and Reemtsma) preferred advalorem taxes, while producers of mainly expensive cigarettes (PM and JTI) preferred specific taxes. In 2003, PM and JTI urged keeping the existing specific tax system, forecasting smuggling increases and revenue decreases should a mixed taxation system be adopted [41]. However in 2004, all TTCs operating in Ukraine (PM, JTI, BAT, Reemtsma and Gallaher) issued a joint statement suggesting the maintenance of the existing taxation structure. They stated that mixed excise system, which did enter into force in 2004, "*meets state revenues interests and provides stable framework for constant development of tobacco industry in Ukraine*" [42]. The statement was caused by the submission of proposals to increase tax rates for the international brands. The threat of a real increase in excise rates seemed to make TTCs forget their previously articulated arguments for a more "beneficial" taxation system. The parliament supported tax rate increases, but the President of Ukraine vetoed the law [43].

In 2005, the Government proposed to increase advalorem excise rates from 7% to 8% and to establish a minimum tax requirement, not in the specific, but in the ad valorem form. Three TTCs (PM, JTI and Gallaher) urged to keep the current tobacco excise structure,[44] again forecasting that smuggling would increase if the current tobacco tax system changed. However BAT did not oppose the new system as it was more beneficial for its brand portfolio and total tax increase was very moderate.

In 2006, the authorities proposed to increase excise rates. TTCs claimed: "*the companies should pay 470 million UAH of taxes more, than with existing rates" *and opposed the changes, again forecasting dire predictions of increases in rates of smuggling [45]. The excise was not increased, and it turned out, that the government actually spent 470 million UAH on fighting alleged smuggling.

In 2007, the Ministry of Finance proposed to raise excise rates and there were no protests from the TTCs[46] as the suggested excise increase was essentially below the inflation level.

**Figure 2:**
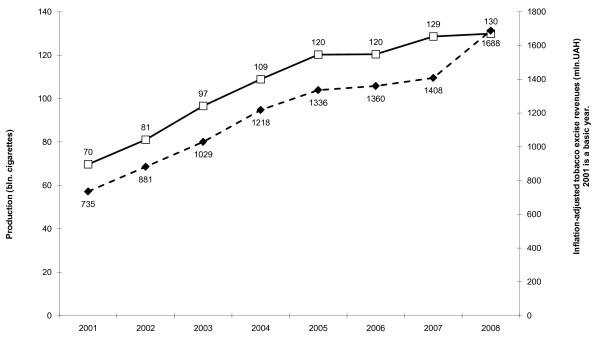
**Cigarette production and revenues in Ukraine in 2001-2008**. Production (bln. cigarettes). Inflation-adjusted tobacco excise revenues (mln.UAH) 2001 is a basic year.

During 2001-2007 tobacco excise changes in Ukraine were mainly determined by the competitive interests of the TTCs. Real (inflation-adjusted) cigarette prices decreased. The lowering cost of cigarettes was one of the main causes of smoking prevalence increase those years [47]. In 2001-2007 the inflation-adjusted revenues increased only in parallel with cigarette production growth (Fig. 2).

### 2008: The excise increase brings one billion to state coffers

In 2008 some MPs proposed essential increases in tobacco excise rates in Ukraine. TTCs opposed these proposals, again predicting the growth of smuggling to Ukraine and a slump in production that allegedly would lead to reduction in revenues. Contrary to the tobacco industry opinion, the parliament approved 1.5-2 fold cigarette excise increase, effective of September 1, 2008. Tobacco industry representatives issued negative comments concerning the bill,[48] repeating arguments that the new excise would lead to cigarette smuggling from Russia. However in July 2008 the BAT representative confirmed that cigarette prices in Russia exceeded Ukrainian prices by 20% [49]. Thus, the recent tax increase in Ukraine just narrowed price differential with Russia, and, according to the tobacco industry claims, narrowing such differential is the main instrument to prevent smuggling. A JTI representative declared, that the government would not receive a planned additional 1.1 billion UAH in revenue from the excise increase, but only 0.5 billion UAH [50]. Eventually the Government had got more than 1 billion UAH additional revenues just within the last four months of 2008, contrary to the industry's forecasts. Cigarette price increases in 2008 were above inflation rates and inflation-adjusted revenue increases exceeded the production growth (Fig. 2).

## Discussion

During all the years of their presence in Ukraine, TTCs have used a range of strategies to influence tobacco taxation with the aim to «*manage taxation to our benefit*» [51]. Their main taxation strategy was rather consistent - «*the lobbying strategy is to keep excise as low as possible*» [40]. and while in the internal documents this strategy is expressed in clear terms, it was not mentioned at all in the TTC's public statements. This paper reveals that self interest has driven TTC's taxation activities in Ukraine.

In those cases where authorities in Ukraine ignored TTC opinion and established higher excise rates than it was suggested by the TTCs, and made tobacco prices to increase above the inflation level, revenues increased. In other countries, governments also often ignored tobacco industry vested interests in taxation policies. In 2001 BAT had to admit that "*the current FCTC process will encourage the momentum towards higher taxation" *and "*governments will increasingly improve their understanding of the economics of the industry and will seek to maximise their overall revenues from the industry" *[52].

The findings of this paper may help to encourage the development of tobacco taxation in line with FCTC principles. Consideration must be given to reversing the strategies of the tobacco industry on tobacco taxation, as these have been shown to be self serving. Increasing tax rates can contribute to the health objectives through reducing tobacco consumption. The main principles of public health-oriented taxation policy can be formulated as: 1) keep excise as high as possible and 2) tax increases need to ensure that retail price of tobacco products increase above rates of inflation.

The findings reveal some contradictions and conflicts between different TTCs as they tried to use the structure of excise as a competition tool. BAT described its competitor's international activities in the following way:[53] «*Philip Morris will continue to campaign for specific taxation in order to support its premium-segment brand focus, selling the concept to governments primarily on the basis of the attributes of consistency and simplicity. Philip Morris's lobbying activities on this issue will be particularly aggressive in new investment markets*». However, PM campaign for specific taxation should not be misinterpreted as a campaign for specific tax rates increase, as real PM approach is to "*increase specific portion of excise tax structures, without increasing total tax incidence" *[15]. While in some countries BAT can oppose such approach it recognizes that «*the optimum balance between specific and ad valorem will depend upon the brand mix of each company*" [14]. In Ukraine conflicts between TTCs over tax structures sometimes became public to hide their real aim to change tax structures without increasing total tax incidence.

Despite the preferences for differences in tax structure reflecting the market position and nature of their brands, in Ukraine TTCs usually ignored these differences, when some initiative on real tax increase emerged, to fight as an industry against it. For public health, the optimum balance between specific and ad valorem will depend on the economic and political situation in the country and there is no single formula to calculate such balance. In Ukraine both pure specific excise (in late 1990s) and mixed excise (in 2008) proved to be able to increase real cigarette prices and revenues. Tobacco control advocates can have different positions with regard to which tax structure better serves public health aims, however, these differences are much less important than total tax incidence increase. If policy makers submit taxation proposals that would result in increasing tobacco prices above inflation, public health advocates should consider supporting such proposals even if proposed tax structure seems not to be optimal one.

TTCs constantly use the threat of smuggling to keep excise low and sometimes this perceived threat prevents authorities from being willing to increase tobacco tax rates. When proposing tax increases, public health advocates should calculate different scenarios concerning cigarette smuggling both into the country and out of the country to ascertain that proposed tax increases will be beneficial for revenues even in the worst (but real) scenario. If tax rates are increased, the impact of such increases on revenues, consumption and smuggling should be monitored to demonstrate inadequacy of tobacco industry forecasts and to encourage authorities to continue to take action to raise tobacco taxes into the future.

Tobacco industry's documents confirm the effectiveness of large cigarette excise tax increases as a policy for governments in their efforts to reduce tobacco use [53]. BAT admitted that «*higher taxes imply higher consumer prices, which will impact sales volumes negatively*» and «*there will be continuing pressure on governments for higher tax rates on cigarettes and other tobacco products, arising from both anti-smoking pressure groups and general state budgetary concerns (with international agencies e.g. World Bank, IMF, WHO, continuing to be influential in pressing for such increases*)» [54].

The tobacco industry recognises that tobacco taxation is a key component in national tobacco control efforts and, if we look to evidence from the Ukraine, has attempted to undermine or subvert its effectiveness. Guidelines for implementation of Article 5.3 of the FCTC on the protection of public health policies with respect to tobacco control from commercial and other vested interests of the tobacco industry [55] should be fully applied to tobacco taxation policy development. According to the Guidelines, as it is not strictly necessary for the authorities to interact with the tobacco industry to effectively regulate tobacco taxes, such interactions should be avoided.

## Conclusions

The FCTC includes provisions both on tobacco taxation policy and on protection of public health policy from vested interests of tobacco industry. This paper provides arguments why tobacco taxation policy should also be protected from vested interests of tobacco industry. The transnational tobacco companies' main taxation strategy is consistent: to keep excise as low as possible. While in their public statements tobacco companies do not disclose this strategy, this aim was clear in the industry documents reviewed in preparing this paper.

Apparent conflicts between TTCs concerning tax structures often hide their real aim to change tax structures for competing interests without increasing total tax incidence.

The study gives grounds for governments, which cares for the population health and filling of the budget, not to allow tobacco companies to influence the tobacco taxation policy. Tobacco tax increases directly benefit governments through increased revenues and they are effective in reducing tobacco use. The reduction in tobacco use, however, is against the vested interests of tobacco companies and they seem to be willing to make considerable efforts to prevent increase of tobacco taxes. The paper reviews arguments and tactics tobacco industry uses to keep taxes as low as possible and provides counter-arguments for developing taxation policy so as to contribute to the health objectives through reducing tobacco consumption.

## Competing interests

The author declares that they have no competing interests.

## References

[B1] World Health Organization WHO Report on the Global Tobacco Epidemic, 2008 - The MPOWER package 2008 Geneva: WHO

[B2] Gilmore A McKee M Tobacco and transition: an overview of industry investments, impact and influence in the former Soviet Union Tob Control 2004 13 136 42 10.1136/tc.2002.002667 15175530 PMC1747859

[B3] Gilmore A McKee M Moving east: how the transnational tobacco companies gained entry to the emerging markets of the former Soviet Union. Part I: Establishing cigarette imports Tob Control 2004 13 143 50 10.1136/tc.2003.005108 15175531 PMC1747849

[B4] Gilmore A McKee M Moving east: how the transnational tobacco companies gained entry to the emerging markets of the former Soviet Union. Part II: an overview of priorities and tactics used to establish a manufacturing presence Tob Control 2004 13 151 60 10.1136/tc.2003.005207 15175532 PMC1747844

[B5] Gilmore AB McKee M Exploring the impact of foreign direct investment on tobacco consumption in the former Soviet Union Tob Control 2005 14 13 21 10.1136/tc.2003.005082 15735295 PMC1747989

[B6] Krasovsky K Andreeva T Krisanov D The economics of tobacco control in Ukraine from the public health perspective 2002 Kiev: Alcohol and Drug Information Centre (ADIC-Ukraine)

[B7] Herter Ulrich "Keynote Speech to the World Tobacco Symposium, Wednesday 22nd September 1993 Ulrich Herter Managing Director Tobacco BAT Industries Plc" 1993 http://tobaccodocuments.org/batco/202232545-2563.html *Bates: 202232545-202232563 *(accessed 5 October 2009)

[B8] Consumer & Regulatory Affairs 1998 Plan Guidelines http://legacy.library.ucsf.edu/tid/vki45a99 Bates 770004490-770004492 (accessed 15 April 2010)

[B9] Campbell R Balbach ED Mobilising public opinion for the tobacco industry: the consumer tax alliance and excise taxes Tob Control 2008 17 351 356 10.1136/tc.2008.025338 18687706 PMC2772174

[B10] Hiilamo H Tobacco industry strategy to undermine tobacco control in Finland Tob Control 2003 12 414 23 10.1136/tc.12.4.414 14660780 PMC1747767

[B11] Szilágyi T Chapman S Tobacco industry efforts to keep cigarettes affordable: a case study from Hungary Cent Eur J Public Health 2004 11 4 223 228 14768787

[B12] Gilmore A Collin J Townsend J Transnational Tobacco Company Influence on Tax Policy During Privatization of a State Monopoly: British American Tobacco and Uzbekistan Am J Public Health 2007 97 2001 2009 10.2105/AJPH.2005.078378 17138915 PMC2040352

[B13] Carter SM Tobacco document research reporting Tob Control 2005 14 368 76 10.1136/tc.2004.010132 16319359 PMC1748115

[B14] Bingham P B.&W. "Key Area Paper. Excise Taxation of Tobacco Products" 1992 http://tobaccodocuments.org/bw/12182942.html Bates: 699138223-699138248. (accessed 15 March 2010)

[B15] PMI, Philip Morris International EEMA Region Situation Assessment http://legacy.library.ucsf.edu/tid/fhl19e00 2500108132/8147. (accessed 17 April 2010)

[B16] Ukraine Business Plan 1992 http://tobaccodocuments.org/batco/301738700-8783.html Bates: 301738700-301738783. (accessed 5 October 2009)

[B17] BAT Industries van Waay A "Ukraine - Marketing" 1992 http://tobaccodocuments.org/batco/301689859-9861.html Bates: 301689859-301689861. (accessed 5 October 2009)

[B18] Van Waay A "Ukraine" 1992 http://tobaccodocuments.org/batco/301689855-9858.html Bates: 301689855-301689858. (accessed 5 October 2009)

[B19] Van Waay A "Ukraine Marketing Section" 1992 http://tobaccodocuments.org/batco/301689862-9864.html Bates: 301689862-301689864. (accessed 5 October 2009)

[B20] "Document WHO184" No date http://tobaccodocuments.org/psc_who/WHO184.html (accessed 5 October 2009)

[B21] 36-page document: CATEGgRy[ Mainline In te'national Brands" http://tobaccodocuments.org/guildford_misc/502584297-4332.html Bates: 502584297-502584332. (accessed 5 October 2009)

[B22] Herter Ulrich Proposed Investment in Ukraine http://legacy.library.ucsf.edu/tid/kxg67a99 Bates 19920827 202209337 (accessed 15 April 2010)

[B23] Johnson Anne Note from Anne Johnson to U Herter enclosing Ukraine briefing note for meeting with president Kravchuk http://legacy.library.ucsf.edu/tid/hgf77a99 Bates 19930205 (accessed 15 April 2010)

[B24] Letter regarding investment in the tobacco industry of Ukraine 1993 http://legacy.library.ucsf.edu/tid/kff77a99 Bates 203463463-203463465. (accessed 15 April 2010)

[B25] "20-page document" No date http://tobaccodocuments.org/guildford_misc/203826349-6368.html Bates: 203826349-203826368. (accessed 5 October 2009)

[B26] R S "New Tobacco Markets" 1992 http://tobaccodocuments.org/batco/203475321-5329.html Bates: 203475321-203475329. (accessed 5 October 2009)

[B27] Deloitte Touch Research of possible ways to increase State budget revenues from tobacco products taxation in Ukraine (in Ukrainian) 1995 Kiev

[B28] Eastern Economist Not Generating Revenue? 1995 http://legacy.library.ucsf.edu/tid/dxz44a99 Bates 700587793 (accessed 17 April 2010)

[B29] Company Plan Ukraine 1997-1999 http://legacy.library.ucsf.edu/tid/zzj55a99 Bates 800193399-800193409 (accessed 18 April 2010)

[B30] Griscom TC "Following Are Highlights of October External Relations Activities and Issues Worldwide" 1997 http://tobaccodocuments.org/rjr/527892328-2335.html Bates: 527892328-527892335. (accessed 5 October 2009)

[B31] Barbaerlitz T "Weekly Highlights, 980427 - 980501" 1998 http://tobaccodocuments.org/pm/2074651753-1756.html Bates: 2074651753-2074651756. (accessed 5 October 2009)

[B32] Note regarding changes and additions to some Ukrainian laws http://legacy.library.ucsf.edu/tid/dxo44a99 Bates 321212901. (accessed 15 April 2010)

[B33] "Pmi Corporate Affairs Weekly Highlights by Region Week of 990531" 1999 http://tobaccodocuments.org/pm/2078787828A-7836.html (est.). Bates: 2078787828A-2078787836. (accessed 5 October 2009)

[B34] "Weekly Highlights for Week of 990712" 1999 http://tobaccodocuments.org/pm/2065537043-7049.html (est.). Bates: 2065537043-2065537049. (accessed 5 October 2009)

[B35] BAT Cross Regional CORA Conference Warsaw 28-30 June, 2000 http://legacy.library.ucsf.edu/tid/alj55a99 Bate 760072518. (accessed 16 April 2010)

[B36] CORA Budget Proposals for 1999 http://legacy.library.ucsf.edu/tid/bxo44a99 Bates 321212896-321212897 (accessed 19 April 2010)

[B37] "Excise and Trade Issue - European Region Quarterly Highlights - Excise 3rd Quarter 1999" No date http://tobaccodocuments.org/batco/325420279-0280.html Bates: 325420279-325420280. (accessed 5 October 2009)

[B38] British-American Tobacco Company Limited Smuggling: Our View 16 February 2000 http://legacy.library.ucsf.edu/tid/iao53a99 Bates 322048732-322048933 (accessed 18 April 2010)

[B39] BAT Cross Regional CORA Conference Warsaw 28-30 June, 2000 http://legacy.library.ucsf.edu/tid/alj55a99 Bate 760072528. (accessed 16 April 2010)

[B40] BAT Ukraine "Section 1 Audit Committee Meeting July 26, 2000" No date http://tobaccodocuments.org/batco/325411887-1891.html Bates: 325411887-325411891. (accessed 5 October 2009)

[B41] Kiryakova A It would be unprofitable to Ukrainian producers to issue expensive and quality products (in Russian) Delovaya nedelya newspaper, 40, November 13-19, 2003 http://www.liga.kiev.ua/smi/show.html?id = 90174 (accessed 9 August 2008)

[B42] Imangulov T Smoke? OK! (in Russian) Profile of Ukraine 2004

[B43] Salivon S Zaitzev A Born dead (in Russian) Business newspaper 2004

[B44] Perets V To whom "to smoke" is expensive (in Russian) From-UA site 2005 http://www.from-ua.com/eco/eco2/424d728c8859e/(accessed 9 August 2008)

[B45] Anonymous The tobacco people are nervous. The discussions around of a nicotinic question have sharply worsened parameters of domestic tobacco industry (in Russian) Business capital newspaper 2006 24 264 http://www.dsnews.ua/markets/art26427.html (accessed 9 August 2008)

[B46] Anonymous Participants of Ukrainian tobacco markets expressed optimistic reaction on the Ministry of Finance initiative to increase cigarette excise duty (in Ukrainian) Seychas newspaper 2007 http://times.liga.net/articles/gs012497.html (accessed 9 August 2008)

[B47] Andreeva TI Krasovsky KS Changes in smoking prevalence in Ukraine in 2001-5 Tob Control 2007 16 202 6 10.1136/tc.2006.019588 17565141 PMC2598499

[B48] Pashkevich O Parliament has agreed to raise the tobacco products duty (in Russian) Akcyz site 2008 http://akcyz.com.ua/news/tobacco/12461.html (accessed 9 August 2008)

[B49] Prilutskiy tobacconist (in Ukrainian) Delo newspaper 2008 http://delo.ua/news/82860/(accessed 19 August 2008)

[B50] Tchebotaryov K Government gives light (in Ukrainian) DailyUA 2008 http://www.daily.com.ua/articles/2/2008-08-67169.html (accessed 19 August 2008)

[B51] "CORA Europe Regional Split - Eastern Europe" No date http://tobaccodocuments.org/batco/321215633-5639.html Bates: 321215633-321215639. (accessed 9 August 2009)

[B52] British-American Tobacco Company Limited Future Business Environment 2001 http://legacy.library.ucsf.edu/tid/qal55a99 Bates 324537933-324538045 (accessed 17 April 2009)

[B53] Chaloupka FJ Cummings KM Morley CP Tax, price and cigarette smoking: evidence from the tobacco documents and implications for tobacco company marketing strategies Tob Control 2002 11 suppl 1 i62 72 10.1136/tc.11.suppl_1.i62 11893816 PMC1766067

[B54] British-American Tobacco Company Limited Bulgaria Marshall Adrian "Future Business Environment -1999" 1999 http://tobaccodocuments.org/batco/325049767-9885.html Bates: 325049767-325049885. (accessed 5 October 2008)

[B55] World Health Organization WHO Framework Convention on Tobacco Control. Guidelines for implementation Article 5.3; Article 8; Article 11; Article 13 2009 Geneva: World Health Organization

